# A New Treatment Algorithm That Incorporates Minimally Invasive Surgery for Pyogenic Spondylodiscitis in the Thoracic and Lumbar Spines: The Results of Its Clinical Application to a Series of 34 Patients

**DOI:** 10.3390/medicina58040478

**Published:** 2022-03-25

**Authors:** Yoichi Tani, Takanori Saito, Shinichiro Taniguchi, Masayuki Ishihara, Masaaki Paku, Takashi Adachi, Muneharu Ando, Yoshihisa Kotani

**Affiliations:** 1 Department of Orthopaedic Surgery, Kansai Medical University, 2-5-1 Shinmachi, Hirakata 573-1010, Japan; saitot@hirakata.kmu.ac.jp (T.S.); tanigutskmsku@gmail.com (S.T.); ishihara0714@yahoo.co.jp (M.I.); kmu.orthopaedics.pak@gmail.com (M.P.); adachita@hirakata.kmu.ac.jp (T.A.); mando@gaia.eonet.ne.jp (M.A.); 2 Department of Orthopaedic Surgery, Kansai Medical University Medical Center, 10-15 Fimizono-cho, Moriguchi 570-8507, Japan; yoshi96k@ja2.so-net.ne.jp

**Keywords:** pyogenic spondylodiscitis, minimally invasive surgery, minimally invasive spinal treatment (MIST), treatment algorithm, percutaneous pedicle screw, lateral lumbar interbody fusion

## Abstract

*Background and Objectives:* Spinal minimally invasive surgery (MIS) experts at the university hospital worked as a team to develop a new treatment algorithm for pyogenic spondylodiscitis in lumbar and thoracic spines. They modified a flow chart introduced for this condition in a pre-MIS era to incorporate MIS techniques based on their extensive experiences accumulated over the years, both in MIS for degenerative lumbar diseases and in the treatment of spine infections. The MIS procedures incorporated in this algorithm consisted of percutaneous pedicle screw (PPS)–rod fixation and transpsoas lateral lumbar interbody fusion (LLIF). The current study analyzed a series of 34 patients treated with prospective selection of the methods according to this new algorithm. *Materials and Methods:* The algorithm first divided the patients into those who had escaped complicated disease conditions, such as neurologic impairment, extensive bone destruction, and the need to be mobilized without delay (Group 1) (19), and those with complicated pyogenic spondylodiscitis (Group 2) (15). Group 1 had image-guided needle biopsy followed by conservative treatment alone with antibiotics and a spinal brace (12) (Group 1-A) or a subsequent addition of non-fused PPS–rod fixation (7) (Group 1-B). Group 2 underwent an immediate single-stage MIS with non-fused PPS–rod fixation followed by posterior exposure for decompression and debridement through a small midline incision (12) (Group 2-A) or an additional LLIF procedure after an interval of 3 weeks (3) (Group 2-B). *Results:* All patients, except four, who either died from causes unrelated to the spondylodiscitis (2) or became lost to follow up (2), were cured of infection with normalized CRP at an average follow up of 606 days (105–1522 days). A solid interbody fusion occurred at the affected vertebrae in 15 patients (50%). Of the patients in Group 2, all but two regained a nearly normal function. Despite concerns about non-fused PPS–rod instrumentation, only seven patients (21%) required implant removal or replacement. *Conclusions:* Non-fused PPS–rod placements into infection-free vertebrae alone or in combination with posterior debridement through a small incision worked effectively in providing local stabilization without contamination of the metal implant from the infected tissue. MIS LLIF allowed for direct access to the infected focus for bone grafting in cases of extensive vertebral body destruction.

## 1. Introduction

Regarding the surgical treatments of spine infections, which involve the thoracic or lumbar spines much more frequently than the cervical spine [1,2,3], the same basic principles apply to spinal and other non-spinal musculoskeletal infections refractory to nonoperative management. These include debridement with surgical removal of infected, necrotic tissue to restore blood supply to the infected area and, in addition, local stabilization [4]. As expected by these principles, an ideal surgical option for spine infections traditionally consists of adequate debridement anteriorly with bone grafting followed by instrumentation and fusion posteriorly, because the targeted pathology most frequently exists in the anterior vertebral column. In the thoracic and lumbar spines, however, the anterior approaches require substantially greater soft tissue injury and morbidity compared to the cervical spines.

With an increasing number of elderly patients with degenerative spine diseases, who tend to have multiple comorbidities, minimally invasive surgery (MIS) has recently developed for spinal stabilization to minimize surgical access-related morbidity. These MIS techniques include percutaneous pedicle screw (PPS)–rod fixation [5] and lateral lumbar interbody fusion (LLIF) with a transpsoas approach [6,7]. The application of these MIS techniques in the management of spine infections has only just begun, for example, PPS techniques for local stabilization [8,9,10,11,12,13,14,15,16,17,18] and LLIF procedures for surgical debridement followed by bone grafting or cage placement [9,16,19,20,21].

Interestingly, original contributions by Deininger et al. (2009) [8] and Fushimi et al. (2012) [22] on pyogenic thoracic and lumbar spondylodiscitis have paved the way for posterior instrumentation alone without anterior debridement as one of the successful MIS treatment options. The two studies indicated the importance of local stabilization, which could eliminate the need for additional surgical debridement and bone grafting in some spine infections. One subsequent report confirmed the clinical utility of PPS–rod fixation alone for a single-level, noncomplicated pyogenic lower thoracic or lumbosacral spondylodiscitis [10].

In light of a recent paradigm shift toward MIS approaches in the field of adult spinal deformity surgery, spinal MIS experts at the university hospital worked on a team, making some modifications to the traditional treatment flow charts for pyogenic spondylodiscitis in the lumbar and thoracic spines. They developed a new treatment algorithm that incorporates MIS techniques based on their extensive experiences accumulated over the years both in MIS for degenerative lumbar diseases and in the treatment of spine infections. The current study has conducted retrospective analyses at a single institution in a series of 34 patients suffering from spine infections treated with prospective selection of the methods according to this new algorithm.

## 2. Materials and Methods

### 2.1. Patients

From August 2017 to December 2020, a total of 34 patients (20 men), aged 36–89 years (mean: 70.9), were treated either conservatively or surgically for pyogenic spondylodiscitis in the thoracic and lumbar spines at the institution. Eighteen patients came to the university hospital directly or through prompt referral from other hospitals, while 16 were transferred from other institutions after hospitalization for an average of 26 days with unsuccessful infection treatment. All patients agreed in writing to undergo the treatments and participate in the study after reading an informed consent form approved by the institutional review board (IRB) (approval number: 2021288).

### 2.2. Clinical Presentation

The clinical symptoms at the initial visit included back pain at the highest rate of 94% (32 of 34 patients), followed by, in descending order, fever > 37.5 °C (44%), radiculopathy (32%), and myelopathy (9%). Infections most frequently affected the lumbar spines in 25 patients (74%), followed by thoracic spines in 4 (12%), lumbosacral spines in 3 (9%), and thoracolumbar spines in 2 (6%). The patients had one or more comorbid diseases including diabetes mellitus in 11 patients (32%), atrial fibrillation/valvular heart disease in 10 (29%), malignant tumors in 5 (15%), liver disease (hepatitis/cirrhosis) in 4 (12%), and atopic dermatitis in 4 (12%).

### 2.3. Diagnostic Workup

All patients underwent diagnostic and follow-up spine MRI scans with a superconducting system (1.5T SIGNA; GE Corp., Milwaukee, WI, USA). The MRI protocol included sagittal and axial T1- and T2-weighted images with a slice thickness of 4 mm, providing a reliable method for diagnosing and delineating the extent of the disease. All patients had spine CT scans via SOMATOM Perspective (Siemens Healthcare K.K., Munich, Germany), which helped distinguish infection from malignancy and demonstrated the extent of bony destruction. Routine laboratory workups included serial assessments of systemic inflammatory markers such as C-reactive protein (CRP) and white blood cell counts.

Prior to the initiation of systemic antibiotic(s) therapy, the blood culture samples were collected, and the pathologic tissue specimens were obtained by CT-guided (14) or fluoroscopically guided (5) percutaneous needle biopsy or surgical biopsy (15) for bacteriological and histological examinations to identify the causative organisms and confirm the diagnosis.

### 2.4. Algorithmic Treatment Selections

**Figure 1:**
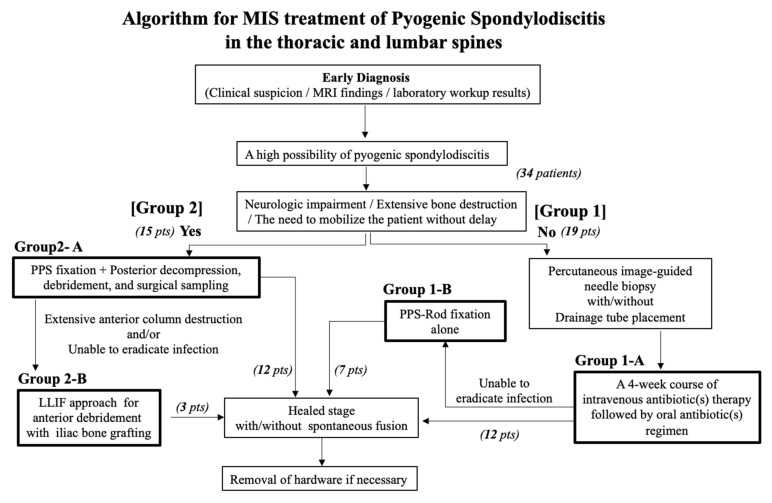
A new treatment algorithm that incorporates minimally invasive surgery for pyogenic spondylodiscitis in the thoracic and lumbar spines. The algorithm first divided the patients into those who had escaped complicated disease conditions, such as neurologic impairment, extensive bone destruction, and the need to be mobilized without delay (Group 1; 19 patients), and those who had complicated pyogenic spondylodiscitis (Group 2; 15 patients). Group 1 patients had image-guided needle biopsy followed by nonoperative treatment (Group 1-A; 12 patients) or a subsequent addition of non-fused PPS–rod fixation (Group 1-B; 7 patients). Group 2 patients underwent an immediate single-stage MIS with non-fused PPS–rod fixation followed by posterior exposure for decompression and debridement through a small midline incision (Group 2-A; 12 patients) or an additional LLIF procedure after an interval of 3 weeks (Group 2-B; 3 patients).

A new decision-making algorithm for the treatment of pyogenic spondylodiscitis in the thoracic and lumbosacral spines (Figure 1), developed by a team of spinal MIS experts, modified a flow chart introduced for this condition in a pre-MIS era [4]. The modified algorithm incorporated recent advances in MIS techniques, such as PPS–rod fixation and LLIF, utilized in degenerative lumbar diseases. The algorithm first divided the patients into those who had escaped complicated disease conditions, such as neurologic impairment, extensive bone destruction, and the need to be mobilized without delay (Group 1), with those who had complicated pyogenic spondylodiscitis (Group 2). Group 1 patients had conservative treatment as a first-line choice, whereas Group 2 underwent an immediate surgical intervention.

### 2.5. Conservative Treatment

Group 1 patients underwent an image-guided percutaneous needle biopsy followed by intravenous administration of linezolid, an oxazolidinone antimicrobial agent, which may have been subsequently switched to specific antibiotic therapy, determined according to the microbial culture and sensitivity test. Linezolid was selected as the post-biopsy antibiotic regimen prior to the establishment of a microbiologic diagnosis, because accumulated evidence indicates that linezolid, although mainly bacteriostatic (inhibitory) rather than bactericidal (lethal) in the mechanism of its action in vitro, has good bone penetration and high activity against Gram-positive organisms, including *Staphylococcus aureus,* whether it is a methicillin-sensitive (MSSA) or methicillin-resistant (MRSA) strain [23], which remains the most common offending organism of pyogenic spondylodiscitis.

In conjunction with an image-guided biopsy, a drainage tube was placed in the vertebral and/or psoas abscess, if found by MRI, for continuous suction drainage for 1–2 weeks. Instead of bed rest, a supportive spinal brace allowed early ambulation for elderly patients who needed mobilization to prevent decubitus ulcers, deep vein thromboses, and pneumonia. Clinical and laboratory responses to such conservative therapies dictated subsequent treatment options. Favorable response allowed for an early switch to an oral antibiotic regimen, whereas serial CRP tests that failed to show a progressive reduction in the values toward normalization guided us to select the isolated PPS–rod fixation as an MIS option.

### 2.6. Surgical Treatment

#### 2.6.1. Isolated PPS–Rod Fixation

Isolated PPS–rod fixation served for treating patients who had increased CRP and/or persistent or worsening posture-related back pain who did not respond to conservative management. After positioning the patient prone with general anesthesia, PPS placements were accomplished into the pedicles of unaffected vertebrae 2 or 3 levels rostral and caudal to the affected vertebrae through small stab wounds made 3–4 cm off midline. In the lumbar spines, a newly developed device called a LICAP (Less Imaging Cannulated Awl and Probe) system (Tanaka Corp., Tokyo, Japan) was utilized for this procedure [24]. The LICAP-assisted PPS–rod fixation, coupled with nerve root monitoring with PPS stimulation [25], facilitated safe screw placement without fluoroscopy or a computer-aided navigation system [26]. In the thoracic spines, however, PPS placements relied on fluoroscopy at the expense of radiation exposure.

#### 2.6.2. PPS–Rod Fixation + Posterior Decompression and Debridement + Surgical Sampling

Group 2 patients with complicated pyogenic spondylodiscitis, with or without impending sepsis, underwent immediate surgical intervention. A single-stage operation was selected with PPS–rod fixation followed by posterior exposure for decompression and debridement through an additional midline incision over the affected vertebral level [12]. The open posterior procedure consisted of detachment of the multifidus muscles unilaterally or bilaterally from the spinous processes and laminae; laminectomies; surgical evacuation of infected and necrotic tissue; collection of tissue samples for cultures and histologic examinations; thorough irrigation with saline; drainage tube placement for postoperative continuous suction drainage. The combination of the two techniques, if performed in a single stage with the PPS–rod fixation first, followed by the posterior exposure, minimized the contamination of the metal implants from the infected tissue, because the PPS technique utilizes the screw trajectory within a compartment of the sacrospinalis muscles isolated from the multifidus muscles located more medially.

#### 2.6.3. LLIF Procedure for Anterior Debridement with Iliac Bone Grafting

An extensive anterior column destruction with potential instability and local kyphosis or unsuccessful eradication of infection by posterior-based surgeries required the next step of minimally invasive transpsoas LLIF procedure according to the decision-making algorithm. Using the XLIF system (NuVasive Inc., San Diego, CA, USA), the infected focus itself was approached, allowing thorough removal of the infected, necrotic tissue under direct visualization followed by iliac bone grafting. Autogenous bone graft has the advantage of offering the patient both osteoconductive and osteoinductive properties with the possible disadvantage of donor-site morbidities. Instead of autograft, a polyetheretherketone (PEEK) cage [15,21], titanium cage [15,21], or porous tantalum cage [27] may provide a better alternative useful for anterior column support. The surgical team, however, maintained the view that an implant should be introduced into an infection-free bed of a recipient’s vertebrae to prevent implant-associated biofilm infections.

### 2.7. Statistical Analysis

The current study used *t*-tests for comparing the time necessary for CRP to return to normal levels between the patients in Group 1 and Group 2, and Fisher’s exact test for comparisons between two qualitative variables with *p* < 0.05 considered significant. For all analyses, SAS JMP software (SAS Institute Inc., Cary, NC, USA) was used.

## 3. Results

### 3.1. Algorithmic Treatment Selections in 34 Consecutive Patients

A total of 34 patients were treated, 12 nonoperatively (Group 1-A), seven with the addition of non-fused PPS–rod fixation (Group 1-B), 12 with immediate surgical intervention consisting of non-fused PPS–rod fixation followed by posterior exposure for debridement and decompression (Group 2-A), and three with additional LLIF for anterior debridement with iliac bone grafting (Group 2-B) as shown in Figure 1 and Table 1. In this series, none of the patients crossed over from the Group 1 treatment to the Group 2 strategy.

### 3.2. Bacteriology Results

**Figure 2:**
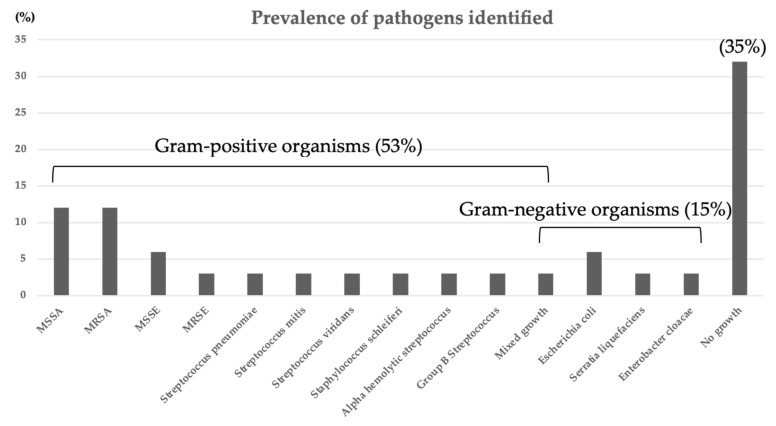
The causative organisms identified by blood culture, percutaneous image-guided needle biopsy, and surgical biopsy. An overall rate of bacteriological identification resulted in 65% (22 of 34 patients). The isolated organisms consisted most commonly of Gram-positive cocci at 81% (18 of 22 patients) such as *Staphylococcus aureus*/*epidermidis* with methicillin-sensitive (MSSA/MSSE (18%)) and -resistant strains (MRSA/MRSE (15%)).

Percutaneous image-guided needle biopsy and/or blood culture identified the causative organisms at a rate of 79% (15 of 19 patients) and surgical sampling at a lower rate of 47% (7 of 15 patients), resulting in an overall bacteriological identification of 65% (22 of 34 patients). The isolated organisms consisted most commonly of Gram-positive cocci at 81% (18 of 22 patients) such as *Staphylococcus aureus*/*epidermidis* with methicillin-sensitive (MSSA/MSSE (18%)) and -resistant strains (MRSA/MRSE (15%)) (Figure 2). The patients in Group 1 tended to have a higher rate of positive culture than those in Group 2, although the difference did not reach statistical significance (*p* = 0.0752) (Table 1). The remaining 12 culture-negative patients continued to have intravenous administration of linezolid followed by an oral antibiotic regimen.

### 3.3. Risk Factors That Necessitated More Complicated Treatment Options

Advanced age (>75 years), positive cultures, MRSA/MRSE infections, comorbid illness, and whether the patients underwent the infection treatment at the university hospital from the beginning or if they had received hospitalized treatment at other institutions before referral did not significantly affect into which treatment groups and categories the patients fell. In contrast, the presence of epidural abscess significantly enhanced the risk of neurologic compromise, resulting in a significantly (*p* < 0.0001) higher incidence in Group 2 than in Group 1 (Table 1).

### 3.4. Follow up and Outcomes

#### 3.4.1. Follow Up

The death of two patients (5.9%) occurred during follow up due to the fact of causes not directly related to pyogenic spondylodiscitis: one patient, an 80 year old woman, who had had Group 1-B treatment, died from septicemia caused by pseudomembranous colitis on postoperative day 21, and the other dialysis patient, a 44 year old man, who had received Group 2-A treatment, died from unknown etiology 9 months postoperatively. Another two patients, who had undergone Group 2-A treatment, became lost to follow up after the last examination on postoperative day 21 and 101, respectively. In the remaining 30 patients, the follow-up period averaged 606 days (range: 105–1522 days) (Table 2).

#### 3.4.2. Clinical, Laboratory, and Radiologic Imaging Results

Of the 12 patients who had immediate surgical intervention because of neurologic impairments (Group 2), all but two regained a nearly normal function. The remaining two patients showed an incomplete return of function. The CRP eventually returned to normal levels in all patients. The CRP values normalized significantly (*p* = 0.00015) earlier for patients in Group 1 than those in Group 2 (Table 2).

Spontaneous interbody fusion occurred at the affected vertebrae in 5 of 18 patients (28%) without direct surgical access to the infected focus (Group 1), while Group 2 treatment achieved the fusion at a significantly (*p* < 0.0078) higher rate of 83% (10 of 12) (Table 2). Of the two patients who failed to achieve the interbody fusion after Group 2-A treatment, one patient developed a rod breakage 2 years postoperatively, requiring revision surgery in which the broken rod was replaced with a new one. The other patient showed extensive vertebral body destruction that required us to move toward the next Group 2-B step of the LLIF procedure with iliac bone grafting to promote rapid healing without collapse.

The patients were explained prior to the surgery about the possibility of long-term mechanical problems and morbidity with non-fused instrumentation, which may require implant removal after the infection was resolved, but only 6 of the 22 patients with PPS–rod fixation (27%) wanted us to remove hardware (Table 2).

### 3.5. Case Presentation

**Figure 3:**
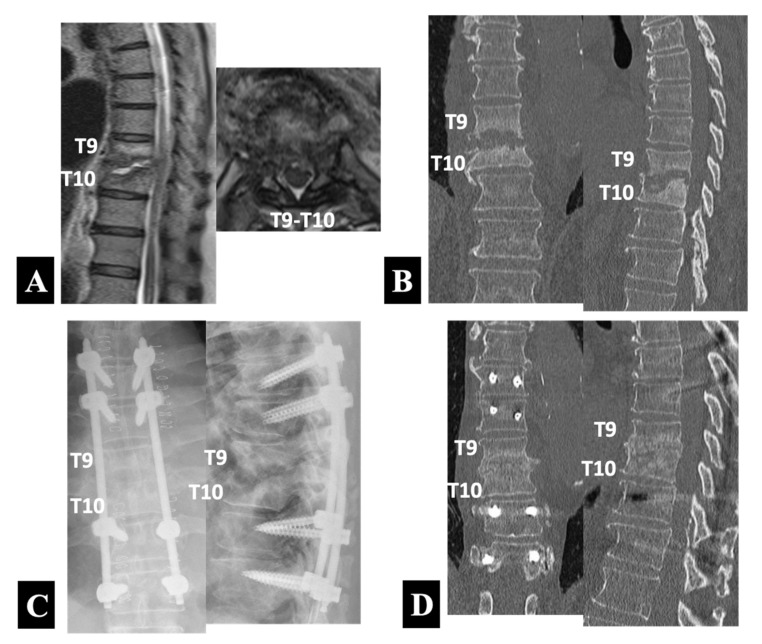
Preoperative sagittal and axial T2-weighted MRIs (**A**) and coronal and sagittal reformatted CT images (**B**) in a 66 year old neurologically intact man with pyogenic spondylodiscitis at T9 and T10. He underwent PPS–rod fixation alone with PPSs placed into the unaffected vertebrae of the T7 and T8 rostrally and the T11 and T12 caudally (Group 1-B) as shown by anteroposterior and lateral plain radiographs taken immediately after surgery (**C**) and coronal and sagittal reformatted CT images 9 months postoperatively (**D**).

Case 1 in Group 1-B (Figure 3):

A 66 year old man presented with severe posture-related back pain and fever. He had been hospitalized to undergo conservative treatment at another institution before being referred to the university hospital for surgery. He had comorbid illnesses such as diabetes mellitus and atrial fibrillation. A medical examination after admission diagnosed his spondylodiscitis as a bloodstream infection from infective endocarditis. The blood culture, which yielded Gram-positive cocci, followed by a sensitivity test determined the use of linezolid as the most specific and least toxic antibiotic agent. The T2-weighted MRI scans (Figure 3A) showed abnormal signal intensities in the peridiscal area of the T9 and T10 vertebral bodies with loss of definition of the endplates, which accompanied abscess formation in the T9–T10 disc space. Consistent with a normal neurologic examination, the MRI scans revealed only a mild cord compression. The CT scans (Figure 3B) demonstrated advanced destruction of the T9 and T10 vertebral bodies involving the endplates.

Because of the absence of neurological symptoms and signs, he underwent PPS–rod fixation alone with PPSs placed into the unaffected vertebrae of the T7 and T8 rostrally and the T11 and T12 caudally. The radiographs taken immediately after the operation (Figure 3C) showed the posterior instrumentation construct. Two months after the operation, he underwent mitral valve repair for infective endocarditis. The CT scans at 9 months follow up (Figure 3D) confirmed a progressive bone formation without an increase in local kyphosis at the affected T9–T10 intervertebral level.

**Figure 4:**
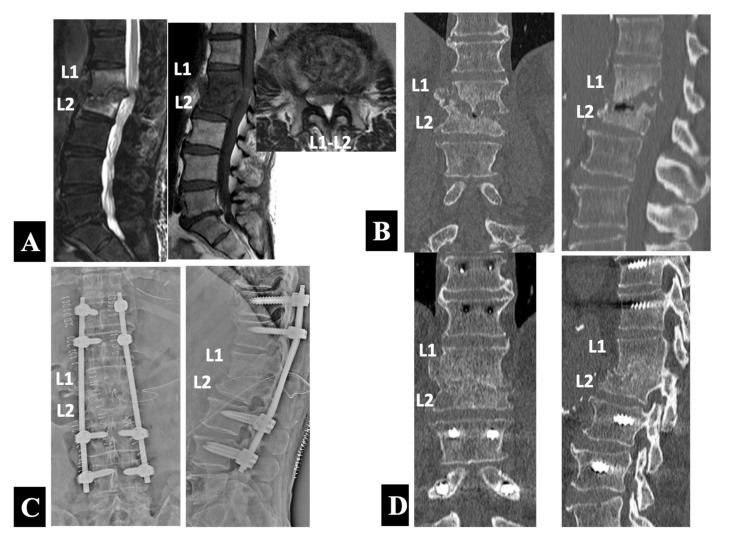
Preoperative sagittal T2- and sagittal and axial T1-weighted MRIs (**A**) and coronal and sagittal reformatted CT images (**B**) in a 77 year old neurologically compromised man with relapsed pyogenic spondylodiscitis at L1 and L2. He underwent a single-stage operation with PPS–rod fixation at the unaffected vertebrae of the T11 and T12 rostrally and the L3 and L4 caudally followed by posterior exposure for decompression and debridement at L1–L2 (Group 2-A) as shown by postoperative anteroposterior and lateral plain radiographs taken immediately after surgery (**C**) and coronal and sagittal reformatted CT images 1 year postoperatively (**D**).

Case 2 in Group 2-A (Figure 4):

A 77 year old man suffered from a relapse of spinal infection, presenting with lower back pain and bilateral lower limb weakness after six months of conservative treatment with a culture-negative result in the CT-guided biopsy. The antibiotic regimen consisted of 4 weeks of intravenous linezolid administration and subsequent oral use of rifampicin in combination with trimethoprim/sulfamethoxazole (TMP/SMX). He had comorbid illnesses such as diabetes mellitus and atrial fibrillation. The MRI scans (Figure 4A) showed typical findings of spondylodiscitis with high-intensity T2 and low-intensity T1 signals of the L1 and L2 vertebral bodies and loss of definition of the endplates. An abscess and/or inflammatory tissues formed in the anterior epidural space with spinal cord compression explained neurological symptoms and signs of the patient. The CT scans (Figure 4B) demonstrated severe destruction of the L1 and L2 vertebral bodies with local kyphosis.

Because of the presence of neurologic compromise, he underwent a single-stage operation with PPS–rod fixation followed by posterior exposure for decompression and debridement at L1–L2. Postoperative radiographs (Figure 4C) showed the posterior instrumentation construct with the PPSs placed into the unaffected vertebrae of the T11 and T12 rostrally and the L3 and L4 caudally. The CT scans 1 year postoperatively and immediately before the implant removal (Figure 4D) revealed bone union at the affected L1–L2 intervertebral level.

**Figure 5:**
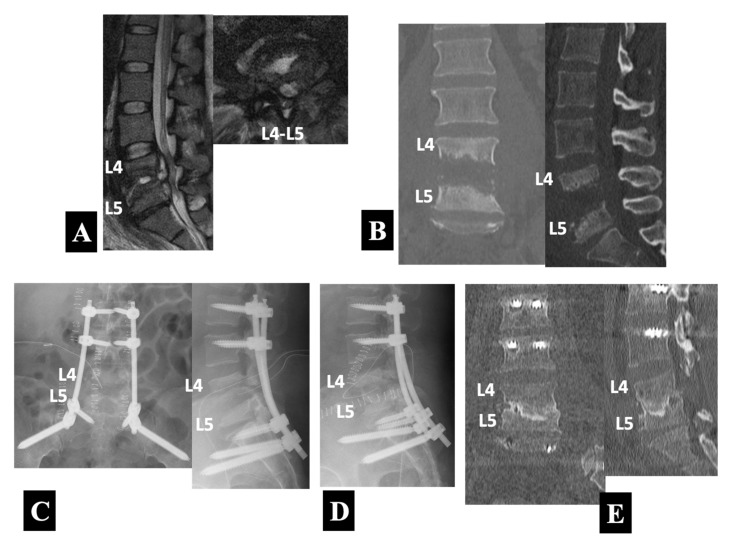
Preoperative sagittal and axial T2-weighted MRIs (**A**) and coronal and sagittal reformatted CT images (**B**) in a 37 year old neurologically compromised woman with pyogenic spondylodiscitis at L4 and L5. She had a two-stage operation separated by an interval of 3 weeks. The first stage of operation, performed urgently, consisted of PPS–rod fixations with the PPSs placed into the unaffected L2, L3, and S1 pedicles and the ilium followed by decompression and debridement through a separate posterior midline exposure at the L4–L5 level as shown by anteroposterior and lateral plain radiographs taken immediately after surgery (**C**). The second stage of surgery utilized an MIS lateral approach for thorough removal of infected necrotic tissues followed by iliac bone grafting at the L4–L5 infected level (Group 2-B) as shown by the lateral radiograph taken immediately after the second surgery (**D**) and coronal and sagittal reformatted CT images one-and-a-half-years postoperatively (**E**).

Case 3 in Group 2-B (Figure 5):

A 37 year old woman was transported to the hospital as a medical emergency due to the fact of difficulty walking with lower back and bilateral leg pain. When the ambulance arrived, the patient turned out to be in septic shock. She had been undergoing dialysis three times a week as a result of diabetic nephropathy. The T2-weighted MRI scans (Figure 5A) showed abnormal signal intensities in the peridiscal area of the L4 and L5 vertebral bodies with abscess formations in the L4–L5 disc space and the anterior epidural space. The CT scans (Figure 5B) demonstrated extensive destructions of the L4 and L5 vertebral bodies.

Intravenous linezolid administration was immediately started. She had a two-stage operation separated by an interval of 3 weeks. The first stage of the operation, performed urgently on the very day the patient was transported, consisted of PPS–rod fixations with the PPSs placed into the unaffected L2, L3, and S1 pedicles and the ilium (i.e., the S2 alar-iliac (S2AI) screw insertion) followed by decompression and debridement with surgical sampling through a separate posterior midline exposure at the L4–L5 level. Postoperative radiographs (Figure 5C) showed the posterior instrumentation construct. The surgical biopsy confirmed pyogenic spondylodiscitis caused by MRSA. The second stage of surgery utilized an MIS lateral approach for thorough removal of infected necrotic tissues under direct visualization followed by iliac bone grafting. The lateral radiograph after the second surgery (Figure 5D) showed an iliac bone graft at the L4–L5 disc space, in addition to the posterior instrumentation construct placed during the first stage of the operation. She received linezolid administration for 4 weeks intravenously and subsequently for 2 weeks orally, followed by 1 year oral use of rifampicin in combination with TMP/SMX. The CT scans at one-and-a-half-years follow-up (Figure 5E) demonstrated bony fusion associated with infection healing.

## 4. Discussion

Modern MIS techniques, such as PPS–rod fixation and the LLIF procedure, have provided considerable benefits in elderly adult spinal deformity surgery by minimizing surgical access-related morbidity. The same surgical principle applies in the management of spine infections, which tend to involve patients with compromised systemic immune function (62% in this series (21 of 34 patients)) (Table 1). Of all spine infections, the lumbar spine ranks the first in incidence and thoracic spine as the second most prevalent location, combining to account for >90% of all cases [1,2,3]. This location of predilection for spine infections makes existing MIS techniques readily available. A team of spinal MIS experts modified a traditional flow chart for decision making in pyogenic spondylodiscitis treatments, developing a new algorithm potentially useful in selecting the MIS techniques for this entity in lumbar and thoracic spines (Figure 1). The current study presented the outcomes of the treatments selected according to this algorithm in a series of 34 patients with this condition.

Systemic administration of the proper antibiotic(s), determined by the culture and sensitivity test, remains to play a major role today in the treatment of spine infections. In this series, an overall rate of bacteriological identification resulted in 65% (22 of 34 patients) (Table 1) that fell within the previously documented values of 36–91% for image-guided biopsy for spinal infections [28]. In culture-negative patients, the current study recommends continuing post-biopsy linezolid administration because of its ability to penetrate bones and high activity against Gram-positive organisms (including MRSA/MRSE) [23], which constitutes the most common pathogens of pyogenic spondylodiscitis (Figure 2). However, one must keep in mind that linezolid may have to be switched to another antimicrobial agent active against Gram-negative pathogens, which linezolid does not include in its spectrum of activity. Of the 34 patients, 19 patients (56%) who had escaped complicated disease conditions, such as neurologic impairment, extensive bone destruction, and the need to be mobilized without delay, in fact, underwent nonoperative management first with antibiotic(s) and a spinal brace, which improved the infection to the healed stage in 12 patients (63%). Interestingly, no patients with successful nonoperative treatment showed an epidural abscess on MRI scans (Table 1).

The remining seven patients, who failed to recover fully with nonoperative management, received PPS–rod fixation (Figure 3), which promoted rapid healing of infections in all patients, including three cases where initial epidural abscesses were resolved spontaneously later on (Table 1). This finding deserves a special mention. Contrary to common belief, the failure of nonoperative treatment does not necessarily imply that the salvage operation for such patients should be surgical debridement of devitalized tissue by approaching the infected focus itself. The PPS–rod fixation alone strategy worked well for patients free of neurologic deficits, as reported by Deiniger et al. [8] and later also by Nasto et al. [10], even in the presence of an epidural abscess as experienced in this series. This result indicates a distinct advantage of this technique over nonoperative treatment in better restoring local stability, which ensures infection healing while allowing for earlier mobilization. Introduction of an implant into the affected vertebrae was avoided to prevent biofilm infections that develop on implant surfaces [29,30], placing the PPSs into the infection-free vertebrae 2 or 3 levels rostral and caudal to the infected vertebrae.

In contrast, initial examinations revealed neurologic impairment in 15 of 34 patients (44%). This higher rate of neurologic deficits in this series than a previously reported incidence of approximately 17% of all vertebral osteomyelitis patients [31] may characterize the patients treated in the tertiary referral spinal center. These neurologically compromised patients, who showed MRI evidence of epidural abscess formation at a significantly (*p* < 0.0001) higher rate than those with normal neurologic functions (Table 1), required immediate surgical intervention to promote neurologic recovery. According to the algorithm, a single-stage posterior-based surgery was selected first, consisting of PPS–rod fixation followed by posterior exposure for decompression and debridement with a small midline incision over the affected vertebral level. As mentioned in the results section and reported previously by Viezens et al. [12], this method has the advantage over the open PS fixations in avoiding the contamination of the metal implants from the infected tissue, because PPS–rod constructs remain within a compartment of the sacrospinalis muscles isolated from the infection site accessed with subsequent surgical exposure. Twelve of the 15 patients (80%) benefited from this posterior-only procedure to heal the infection (Figure 4).

The amount of anterior column destruction influenced the direction of subsequent treatment: the remaining three patients (20%) had severe destruction of the affected vertebral bodies, which placed the patients at higher risk for segmental instability and kyphosis. In this case, the algorithm led us to an iliac bone grafting with the transpsoas LLIF procedure 3 weeks after the first-stage, posterior-based surgery with a subsequent proper antibiotic regimen (Figure 5). This staged surgical strategy postponed the bone grafting for 3 weeks for the second stage, unlike a single-stage anterior debridement and the bone grafting [9,16,19,20] or cage placement [21] as reported previously with MIS lateral approaches. The strategy provided a clean, infection-free bed of recipient vertebrae for iliac strut in conjunction with cancellous autograft, offering the best prospects for promoting bone union. In fact, all of the three patients achieved a solid interbody fusion (Table 2).

Finally, the issue of minimally invasive instrumentation without fusion should be briefly addressed. As mentioned above regarding the posterior instrumentation, the PPSs were placed only into the infection-free vertebrae 2 or 3 levels above and below the infected vertebrae, which were skipped over to prevent implant-associated biofilm formation that may preclude the successful eradication of the infection. Consequently, PPS–rod instrumentation covered the adjacent non-fused segments next to the affected level, raising clinical concerns about the risk of long-term implant failures even after fusion occurred between the affected vertebrae. Therefore, the patients were informed preoperatively about the potential need for surgical removal of the implant after infection healing. Despite the concern, however, only 6 of 34 patients (18%) wanted the implant removed (Table 2) and another one patient developed rod breakage requiring surgical revision to replace it with a new rod. The relatively low rate of revision surgery for implant removal counters the commonly held notion that all the vertebrae included in the instrumentation area need to be fused. Similarly, Nasto et al. (2014) [10] treated 12 patients with noncomplicated (i.e., known infectious agent, no neurologic compromise, and preserved spinal stability) pyogenic spondylodiscitis in the thoracic or lumbar spine using PPS–rod instrumentation that involved percutaneously instrumented non-fused segments one level above and below the infected vertebrae. Interestingly, they reported that none of the patients wanted the implant removed at 9 months follow up. More recently, Lau and Chou (2017) [32] compared the rates of implant failure requiring reoperation between 21 patients with percutaneously instrumented non-fused segments and 32 patients with open instrumented fused segments. Those patients underwent either mini-open or open posterior thoracic corpectomies with cage reconstruction followed by supplemental pedicle screw instrumentation 2 or 3 levels above and below the corpectomy site for a variety of disorders including spinal infections. The comparative results showed a similar rate of posterior implant failure between the two groups with 8.3% in the former non-fused cases and 18.2% in the latter (*p* = 0.438) at 2 year follow up. These data together with the experience in this series suggest the possibility of PPS–rod instrumentation without fusion as a viable option in appropriate clinical settings such as the presence of anterior column support and older, physically less active, lower demanding patients or patients with short life expectancies [33]. This does not hold true, of course, unless the clinical benefits outweigh the risk of implant failure.

## 5. Study Limitations

The current study had several limitations. Firstly, the number of patients fell short of that required to validate the algorithm, which means further study on a larger series is required. Secondly, although the algorithm provides a simplified overview for ease of use, it may carry the risk of oversimplification considering various other factors that play an important role in the decision-making process for treating this serious disease. Thirdly, a new algorithm utilized in the current study adheres rigidly to two traditional general principles: (1) the priority given to nonoperative management over surgical intervention; (2) a safety concern regarding the possibility of implant-associated biofilm infections caused by its placement in the infection site. The algorithm, therefore, does not include the recently reported option of an early MIS for anterior column support with cage placements to prevent loss of lumbar lordosis. Fourthly, a lack of technical novelty in each MIS procedure employed in this algorithm may reduce the impact of this study. Nonetheless, the algorithm will provide the basis of strategy for application of various MIS techniques in the management of spine infections, which has just begun.

## 6. Conclusions

The current study analyzed the results of pyogenic spondylodiscitis in thoracic and lumbosacral spines in a series of 34 patients treated with prospective selection of the methods according to a new algorithm that incorporates MIS techniques. The algorithm first divided the patients into a non-complicated group (*n* = 19) (i.e., no neurologic impairment, no extensive bone destruction, and no need to be mobilized without delay) and a complicated group (*n* = 15). Non-fused PPS–rod placements into infection-free vertebrae alone or in combination with posterior debridement through a small incision worked effectively in providing local stabilization without contamination of the metal implant from the infected tissue. MIS LLIF allowed for direct access to the infected focus for bone grafting in cases of extensive vertebral body destruction.

## Figures and Tables

**Table 1:**
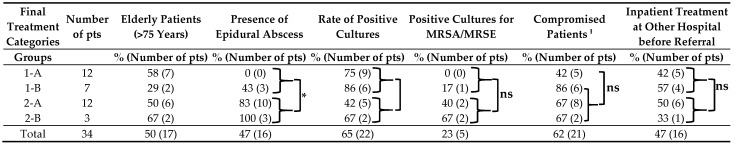
Risk factors that necessitated more complicated treatment options.

^ⱡ^ Compromised patients included those with diabetes, systemic steroid use, malignant neoplasms, hepatic cirrhosis, or dialysis. * *p* < 0.0001. ns, not significant; pts, patients.

**Table 2:**
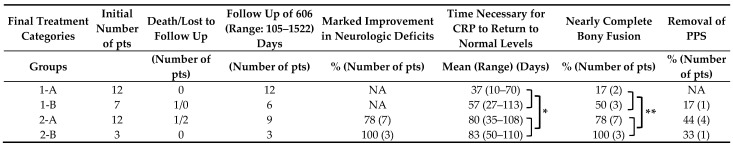
Evaluation of outcome.

* *p* = 0.00015; ** *p* = 0.0078. NA, not applicable; pts, patients.

## Data Availability

Not applicable.
